# Clinical observation and study of local hyperthermia for treating plantar warts: A pilot study with 38 patients

**DOI:** 10.3389/fmed.2023.1087659

**Published:** 2023-01-26

**Authors:** XiaoLi Chen, Yan Xu, Li Hu, AiJun Chen

**Affiliations:** Department of Dermatology, The First Affiliated Hospital of Chongqing Medical University, Chongqing, China

**Keywords:** plantar warts, clinical observation study, local hyperthermia, warts disease, adverse event

## Abstract

Warts are benign lesions caused by infection of the keratinocytes by the human papillomavirus (HPV). There is still no consensus on the standard treatment for plantar warts, and the various treatments (both destructive and non-destructive) have variable efficacy with a long list of shortcomings, such as higher recurrence, pain, and scarring. Local hyperthermia was considered a safe, effective, and promising therapy in the treatment of plantar warts. After getting approval from the hospital’s ethics committee, the present study was designed to assess the clinical efficacy of local hyperthermia in the treatment of plantar warts. A total of 38 patients were enrolled in the study, and all patients received a standard regimen in a 5-week schedule, with local 45°C treatment for 30 mins on days 1, 2, 3, 14, 15, 22, 29, and 36. Of the 38 patients, complete resolution of the warts was observed in 13 (34.2%), 8 (21.1%) achieved partial remission, and 17 (44.7%) revealed poor response to the treatment. Patients were followed up for at least 3 months, and there was no recurrence of lesions in the 13 clinically cured patients at the last follow-up. The findings in the current study demonstrate that local hyperthermia is a safe, effective, and promising therapy for the treatment of plantar warts.

##  1. Introduction

Plantar wart is a common skin disease caused by human papillomavirus (HPV) foot infection. It is estimated that up to 22% of the general population and 33% of primary school children have cutaneous warts (1). Although spontaneous regression is often observed in plantar warts, 2% of the general population who suffer from pain and limitations to sports and daily activities seek medical care for plantar warts annually (2). However, there is still no consensus on the standard treatment for plantar warts, and average cure rates for many treatment options varied in a review study as follows: cryotherapy (45.61%), laser (79.36%), topical antivirals (72.45%), and intralesional immunotherapy (68.14%) (3). The typical adverse effects, such as pain, bleeding, secondary infection, and ulceration, were noted in patients treated with these therapies (4). Some studies have reported that local hyperthermia is highly effective in treating plantar warts, with an average 58.8% clearance rate and only a few mild adverse reactions, such as a burning sensation (5). To the best of our knowledge, there is limited literature reporting on the effect of local hyperthermia on warts. Therefore, the present study was designed to assess the clinical efficacy of local hyperthermia in the treatment of plantar warts and to investigate the potential factors affecting the treatment responses.

## 2. Materials and methods

A total of 38 outpatients with plantar warts were consecutively enrolled in the prospective study, which was conducted in The First Affiliated Hospital of Chongqing Medical University from January 2021 to July 2021. The institute ethics committee approved the study protocol, and informed consent was obtained from each patient according to institutional regulations following the Declaration of Helsinki. The diagnosis of plantar warts was made independently by two dermatologists based on clinical presentation. If they failed to reach an agreement on the diagnosis, dermatoscopy and histopathology were performed for further confirmation. In addition, what needs to be pointed out is that histopathology is further used in patients with multiple lesions. The main inclusion criteria were the presence of plantar warts and being willing to receive local hyperthermia treatment. Patients with severe systemic disease or immunosuppressed status, such as HIV infection, were excluded. Demographic data and clinical characteristics (e.g., age, sex, course of disease, number of warts, and maximum diameter of warts) were collected from all patients. In addition, pre-and post-treatment photographs of the lesions were taken in all cases, and the adverse reactions, recurrence rate, and clinical efficacy were recorded. Follow-ups were done at least monthly after the treatment by various means, including outpatient visits, phone calls, and WeChat. We regarded the evaluation of the cure rate as the primary outcome. According to Huo et al. (6), complete resolution of all warts, including distant warts, is classed as clinically cured, while partial remission is defined as a 50% response in the lesions *in situ* and distant lesions. The treatment was considered to have failed if no response was observed in any of the lesions. To investigate the potential factors affecting the treatment responses, the patients were classified into two groups according to their response to local hyperthermia.

**FIGURE 1:**
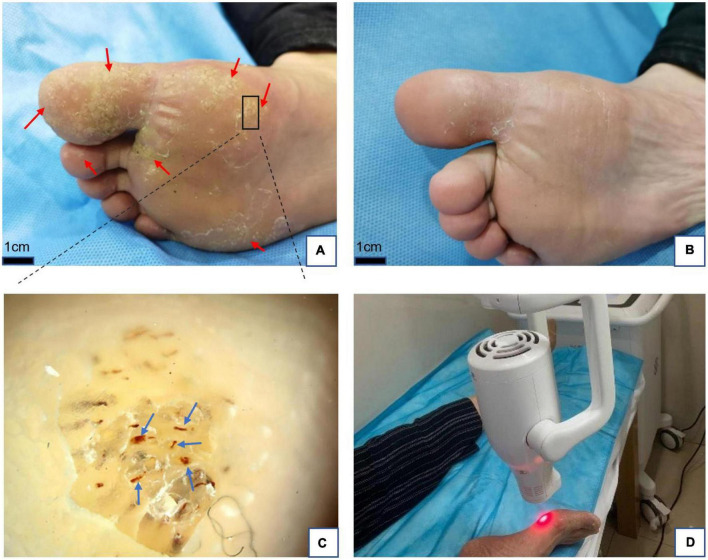
**(A)** Plantar warts (red arrows) before treatment. **(B)** Five weeks after treatment with local hyperthermia (complete resolution). **(C)** Dermoscopic findings in warts, red dots, and linear vessels (blue arrows) on the papilliform surface. Dermoscope 50× magnification. **(D)** Clinical application of local hyperthermia.

The hyperthermia device (Patent No. ZL200720185403.3, China Medical University, China), which is used to generate infrared temperature acted locally on lesions, has been systematically described in previous studies (Figure 1D) and a standard technique described previously was followed to treat the patients (6, 7). Briefly, all patients received a standard regimen in a 5-week schedule, with local 45°C treatment for 30 mins on days 1, 2, 3, 14, 15, 22, 29, and 36. If the patient presented with multiple lesions, we regarded the biggest wart as a targeted lesion. A few patients who did not respond to the complete therapy of hyperthermia were adjusted to other treatments.

Dermoscopic examination of the studied warts was performed using a dermoscope (Dermat Image System, Dermat, Beijing, China) in our Department of Dermatology by physicians who had received specialized training from the Chinese National Telemedicine and Connected Health Center.

Statistical analysis was performed using the SPSS software, version 20 (Chicago, IL, USA). Dichotomous variables from the two groups were analyzed using the Chi-square test, and continuous variables were examined using the Mann–Whitney U test. The significance of the obtained results was judged at the 5% level (*P* ≤ 0.05).

## 3. Results

A total of 38 patients with plantar warts who met the inclusion criteria were enrolled in this study, including 22 males (57.9%) and 16 females (42.1%), ranging from 16 to 70 years, with an average age of 36.2 years. Of these cases, 19 patients had not received any treatments with their initial plantar wart diagnosis, and 19 had undergone CO_2_, laser, cryotherapy, or other treatments. The median time between lesion onset and local hyperthermia was 12 months (1–120 months). The subjects comprised 14 patients who presented with a single lesion and 24 patients with multiple lesions. The number of warts ranged from 1 to 60 with a median of 10, and the maximum diameter ranged from 2 to 45 mm.

**FIGURE 2:**
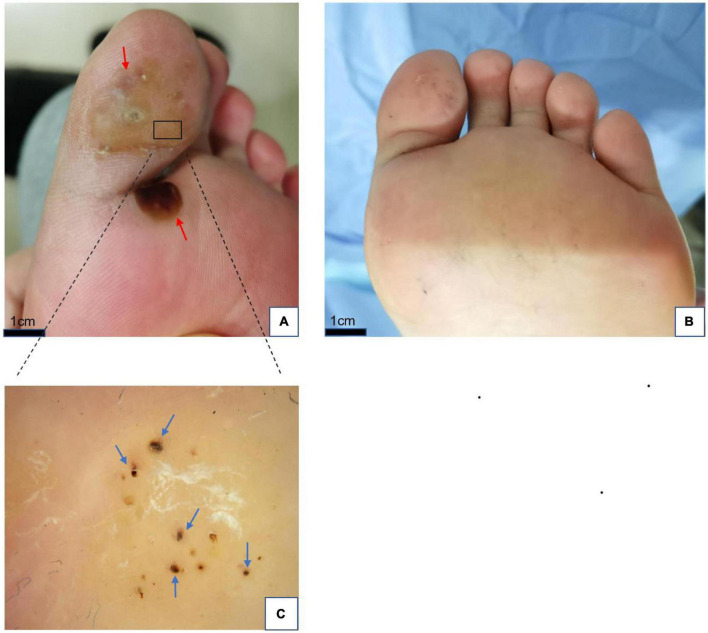
**(A)** Plantar warts (red arrows) before treatment. **(B)** Five weeks after treatment with local hyperthermia (complete resolution). **(C)** Dermoscopic findings in warts; yellowish structureless with bleeding streaks and spots (blue arrows) on the papilliform surface. Dermoscope 50× magnification.

**FIGURE 3:**
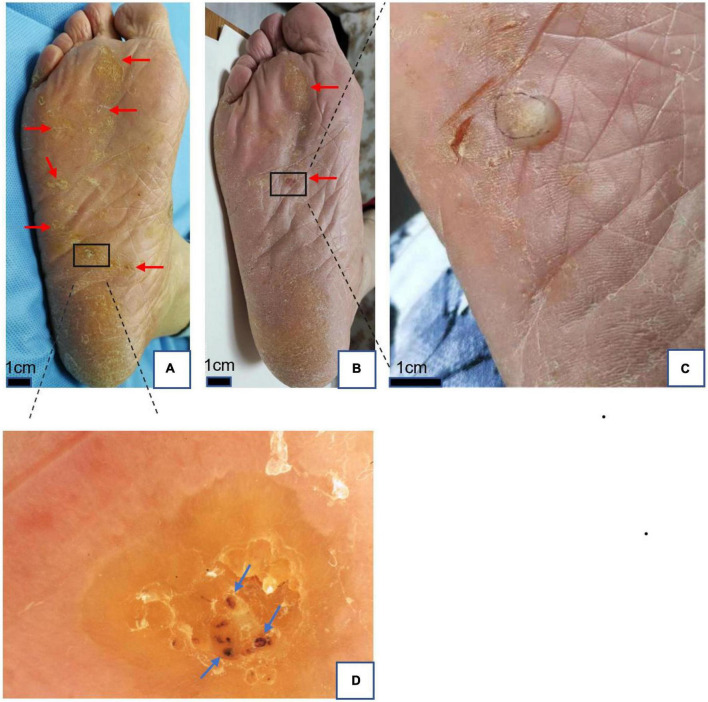
**(A)** Plantar warts (red arrows) before treatment. **(B)** Two months after treatment with local hyperthermia (partial remission). **(C)** Bullae at targeted warts after treatment. **(D)** Dermoscopic findings in warts; yellowish structureless with bleeding streaks and spots (blue arrows) appearance on the papilliform surface. Dermoscope 50× magnification.

Before treatment, all target warts that were not diagnosed were examined using a dermoscope with a fixed magnification of 50×. We found that the background of the wart is usually grayish white, pale yellow, or normal skin color. Tiny black and red dots or red linear vessels may be visible at the papilliform surface of the wart, and these represent thrombosed dilated capillaries (Figures 1C, 2C, 3D). In addition, hyperkeratosis (interrupted skin lines) can also be seen.

All patients completed treatment, and no patients were lost to follow-up. The average follow-up time was 4.6 months (4–7 months). Of the 38 patients, complete resolution of the warts was observed in 13 (34.2%) (Figures 1, 2), while 8 (21.1%) achieved partial remission (Figure 3), and 17 (44.7%) demonstrated poor response to treatment. According to the response to treatment, the patients were classified into response and non-response groups, and there were no statistically significant differences in baseline demographic and clinical characteristics between the two groups (Table 1). A total of 15 patients complained of a tolerable mild burning sensation during the procedure, and other side effects were barely observed. Bullas appeared at targeted warts in two patients (Figure 3D). There was no recurrence of lesions in the 13 clinically cured patients at the last follow-up.

**TABLE 1:** Demographics and clinical characteristics of 38 patients with plantar warts: A subgroup comparison.

Variable	Response group (*n* = 21)	No response group (*n* = 17)	*P*-value
Age median (QS)	32 (26.5, 43.5)	31 (28.5, 41)	0.769
Gender (male/%)	11/52.4%	11/64.7%	0.444
Duration median (QS)	12 (3, 24)	12 (3.5, 78)	0.174
Number median (QS)	5 (1, 26.5)	3 (1, 6.5)	0.366
Diameter median (QS)	8 (4, 15)	10 (4, 11.5)	0.894
Adverse event (yes/%)	8/38.1%	9/52.9%	0.360
Previous treatment (yes/%)	9/42.9%	10/58.8%	0.211

## 4. Discussion

The results of the current study demonstrated that local hyperthermia is a safe, effective, and promising therapy for the treatment of plantar warts. However, the cure rate in this study was 34.2%, which was lower than that of 53.57% reported by Huo et al. (6) with a similar treatment regimen for plantar warts. The reason for this discrepancy is unclear but may be related to HPV subtypes, immunity, or insufficient sample size. Moreover, it is worth mentioning that previous treatments in some patients may play an important role in influencing the therapeutic effect. In our series, the cure rate in 19 patients who had previously received treatment for plantar warts was 26.3%, whereas the cure rate in 19 patients who had not previously been treated was 42.1%. Nevertheless, there was no statistically significant difference between the two groups. Sample size and selection bias may contribute to the result of no statistical difference. The study will continue to recruit patients to determine which factors affect the efficacy of the treatment.

In contrast, we noted that the recurrence rate was quite low, and the untargeted warts were completely dissipated in some patients who presented with multiple warts. Similar features of hyperthermia have been reported in previous studies (8). In contrast to hyperthermia, conventional treatments are highly effective for visibly infected lesions, but invisible HPV infection leads to a high recurrence rate. Although the mechanisms of hyperthermia for the treatment of viral infection are not clear, these features of hyperthermia indicate that it could promote the establishment of a robust HPV-specific immune response to clear all infected tissue. Studies have depicted that hyperthermia at 42–45°C can promote migrational maturation of Langerhans cells, improve their antigen-presenting ability, and clear HPV-infected keratinocytes by establishing a specific cellular immune response (9). In addition to treating plantar warts, local hyperthermia has also been reported to have achieved surprising results in treating giant condyloma acuminatum, immunodeficient patients with extensive warts, and extensive flat warts (10–14).

## 5. Conclusion

In summary, given that many of its mechanisms and appropriate conditions are still unclear, local hyperthermia was shown to be safe and relatively effective in treating plantar warts as a recently developed therapy. Further studies will require a larger sample size and rigorous experimental design to demonstrate the effects of previous treatments in patients with plantar warts.

## Data availability statement

The raw data supporting the conclusions of this article will be made available by the authors, without undue reservation.

## Ethics statement

The studies involving human participants were reviewed and approved by the First Affiliated Hospital of Chongqing Medical University. The patients/participants provided their written informed consent to participate in this study. Written informed consent was obtained from the individual(s) for the publication of any potentially identifiable images or data included in this article.

## Author contributions

XLC participated in the conception and drafted the manuscript. LH and AJC contributed to the study design and revision of the manuscript. YX was responsible for the acquisition of data and the follow-up examinations in the hospital. All authors contributed to the article and approved the submitted version.
